# Texas State Medical Association

**Published:** 1897-03

**Authors:** 


### Society Notes.

### Texas State Medical Association.

*To Members of Texas Medical Association:*

Members intending to favor the Section of OPHTHALMOLOGY, OTOLOGY, ect., with papers, are requested to send the *title* of their papers to the undersigned before March 15, that it may be printed in the preliminary announcement and program for the next meeting at Paris, commencing April 27.

VARD HULEN, M. D., Chairman.

ENNIS, TEXAS, Feb. 1, 1887.

DEAR DOCTOR:—Allow me to call your attention to the meeting of the Texas State Medical Association which will convene in its 29th annual session in Paris, Texas, April 22 and 25th inclusive. The committee of arrangements are making every necessary preparations for the success of the meeting.

A number of papers from some of our most eminent medical men, on subjects of interest and practical value to the busy practitioner have been promised.

The bill to regulate the practice of medicine in Texas as recommended by the State Medical Association at Fort Worth last April will very likely be enacted into a law by the present legislature, and if it is, The State Medical Association at this session will be expected to name those whom they will recommend to the governor for members of the State Medical Examining Board.

Under the operation of the law of our Association as amended at Fort Forth there will be more time given to section work and the discussion of scientific subjects than has heretofore been devoted to them at our annual meetings, which will serve to enhance this interest very much to the earnest seeker of medical and scientific truth,

The evidences at this time are such as to justify the opinion that the State Medical Association is beginning an era of usefulness and success, such as merits and should receive the cordial support of the medical profession of the State. We not only invite you to attend the meeting of the Texas State Medical Association at Paris, Texas, April 22 to 25, 1879, and give to our noble cause the encouragement and support of your presence and your best efforts in behalf of organized medicine in Texas, but we ask that you enlist your medical neighbors and secure their attention and share with us the pleasures and benefits of the great work now engaging the medical thought of this age of rapid progress.

Yours truly, J. C. LOGGINS, M. D.,
Pres. Tex. State Med. Ass'n.

---

FORT WORTH, TEXAS, Feb. 1, 1897.

MY DEAR DOCTOR:—The time for the annual meeting of the State Association is at hand. The Surgical section must not be lacking its usual scientific interest. Will you not contribute to the entertainment, enjoyment and profit of the coming meeting by sending AT ONCE the title of a paper to be presented by you, on some surgical subject, to Dr. A. C. Scott, Secretary, Temple, Texas, or to your chairman?

BACON SAUNDERS, Fort Worth, Tex.

---

**The Fifth Annual Meeting** of The Tri-State Medical Society of Iowa, Illinois and Missouri, will meet in St. Louis, April 6th, 7th and 8th, 1897. A large number of valuable papers will be read. Dr. Joseph Price, of Philadelphia, will hold the Surgical Clinic; Dr. James T. Whittaker, of Cincinnati, the Medical Clinic, and Dr. Dudley Reynolds, Ophthalmic Clinic. Dr. G. Frank Lydston, of Chicago, will entertain the members with an original story during one of the evening sessions. The officers are- A. H. Cordier, M. D., Pres't, Rialto Bldg, Kansas City; Hugh T. Patrick, M. D., 1st Vice-Pre's, Chicago; H. C. Eschbach, M. D., 3d Vice-Pres't, Albia, Ia.; G. W. Cale, M. D., Sec'y, 4403 Washington Boulevard, St. Louis; C. S. Chase, M. D., Treasurer, Waterloo, Iowa.

